# MTLRP genetic polymorphism (214C>A) was associated with Type 2 diabetes in Caucasian population: a meta-analysis

**DOI:** 10.1186/1476-511X-13-124

**Published:** 2014-08-05

**Authors:** Li-Li Chen, Song-Mei Han, Fei-Fei Tang, Qiang Li

**Affiliations:** 1 Department of Endocrinology, The Second Affiliated Hospital of Harbin Medical University, NO. 246, Xuefu Road, Nangang District, Harbin, Heilongjiang Province 150086, P.R. China

**Keywords:** *MTLRP*, Polymorphism, Type 2 diabetes, Meta-analysis

## Abstract

**Background:**

Previous studies reported the relation between *MTLRP* genetic polymorphism and type 2 diabetes, however, the conclusion were conflicting. In the present study, we performed a meta-analysis to reveal this association.

**Methods:**

Literature retrieval, selection and assessment, data extraction, and meta-analyses were performed according to the RevMan 5.0 guidelines. In the meta-analysis, we utilized random-effect model or fixed-effect model to pool the Odds ratio (OR) according to the test of heterogeneity.

**Results:**

A total of nine case–control studies included 4460 type 2 diabetes patients and 4114 healthy control subjects were analyzed. We did not found association between the *MTLRP* polymorphism and type 2 diabetes risk in the overall population (CC *vs* CA + AA: OR = 1.02; 95% CI: 0.89-1.17, P = 0.77; A *vs* C: OR = 1.02; 95% CI: 0.84-0.96, P = 0.62). However, in subgroup analyses stratified by ethnicity, we found significant association of MTLRP polymorphism with type 2 diabetes in Caucasians (CC *vs* CA + AA: OR = 1.27; 95% CI: 1.02-1.57, P = 0.03; A *vs* C: OR = 0.74, 95% CI: 0.60–0.91, P = 0.005).

**Conclusion:**

The *MTLRP* polymorphism was associated with type 2 diabetes in Caucasians.

## Introduction

The prevalence of type 2 diabetes is rapidly increasing and has become one of the most common chronic diseases worldwide [1,2]. Since type 2 diabetes is associated with an increased incidence of cardiovascular disease and long-term mortality, it imposes a significant economic burden on healthcare worldwide [3,4]. Recently, genetics were considered to be the main factor contributing to the development and progression of type 2 diabetes [5,6]. Therefore, the identification of genetic variants involved in individual susceptibility to type 2 diabetes can assist our understanding of the underlying disease process, and help to develop novel therapies.

Ghrelin is a 28 amino acid peptide with an n-octanoylated serine 3 residue primarily expressed in the X/A-like oxyntic cells of the stomach but also in the hypothalamus and pancreas [7-10]. Ghrelin was discovered as the endogenous ligand of the growth hormone secretagogue receptor (GHSR). The peptide stimulates growth hormone release and appetite [11,12]. Administration of ghrelin in rodents induces weight gain by a reduction in fat utilization [13], and ghrelin stimulates appetite and regulates the weight balance through the GHSR by activation of neuropeptide Y (NPY) and agouti related peptide (AGRP) containing neurons in the hypothalamus [14,15]. *MTLRP* is a gene coding Ghrelin peptide and has been considered as a candidate gene for type 2 diabetes. The human *MTLRP* gene is located on chromosome 3 and consists of four exons and three introns. Several single nucleotide polymorphisms (SNPs) have been described within this gene. One missense polymorphism, 214C>A at codon 72 with Met replacing Leu, is outside the region where the mature ghrelin product is encoded and its functional significance remains largely unknown. Recently, there were many genetic studies reported the association between this polymorphism and type 2 diabetes. However, evidence from these studies remains conflicting, rather than conclusive. Although the previous meta-analysis performed by Liao et al. in 2013 [16] has reported on the relation between this polymorphism and type 2 diabetes, it involved less literatures and had lower power. Furthermore, three important studies [17-19] were not included in Liao et al.’s studies. Therefore, for the present study, we have collected almost all published case–control studies to perform an update meta-analysis to further investigate the association between this SNP and risk for type 2 diabetes.

## Materials and methods

### Literature collection and screening

We searched and identified literatures in PubMed, EMBASE, ISI Web of Science, Wangfang in China and CNKI databases using the terms "MTLRP " or "ghrelin" or "preghrelin" and "polymorphism" or "SNP" or "Leu72Met " or "214C>A" and "type 2 diabetes".

### Inclusion and exclusion criteria

The inclusion criteria was as follows: 1) the clinical research of direct comparison of MTLRP polymorphism between Type 2 diabetes and health control subjects without any restriction on language or publication year; 2) Type 2 diabetes patients were diagnosed according to WHO criteria or American Diabetes Association criteria without any restriction on age or racial; 3) the design was case–control study; and 4) genotypes were clearly reported or could be calculated.

### Literature quality assessment and data extraction

Two independent reviewers (LLC and SMH) carried out the literature filtering and quality assessment. We excluded literatures obviously does not meet the inclusion criteria and duplicated publications. If inconsistencies existed, we resolved it through discussion. The Cochrane Handbook 5.0 Quality evaluation criteria were utilized to evaluate methodological quality of included studies. For each study, the following characteristics were extracted: the first author’s last name, year of publication, country of origin, the numbers and age of cases and controls.

### Data analysis

We utilized RevMan 5.0 software which was provided by the Cochrane Collaboration to perform the meta-analysis and to merge the OR values. We directly used Q-test and I^2^ test to examine the heterogeneity between each study. The OR value was utilized to evaluate the relationship between the *MTLRP* polymorphism and Type 2 diabetes. By heterogeneity test, if P > 0.05, we select the fixed effect mode1, and if P <0.05, we select the random effect mode1 to merge OR. P <0.05 was considered as significant difference. Analysis of sensitivity includes the difference of point estimation and confidence intervals of the combined effects value at a different model, to observe whether it changes the result. To test the publication bias, we utilized the RevMan 5.0 statistical software to make the funnel plot.

## Results

### Literature screening

**Figure 1:**
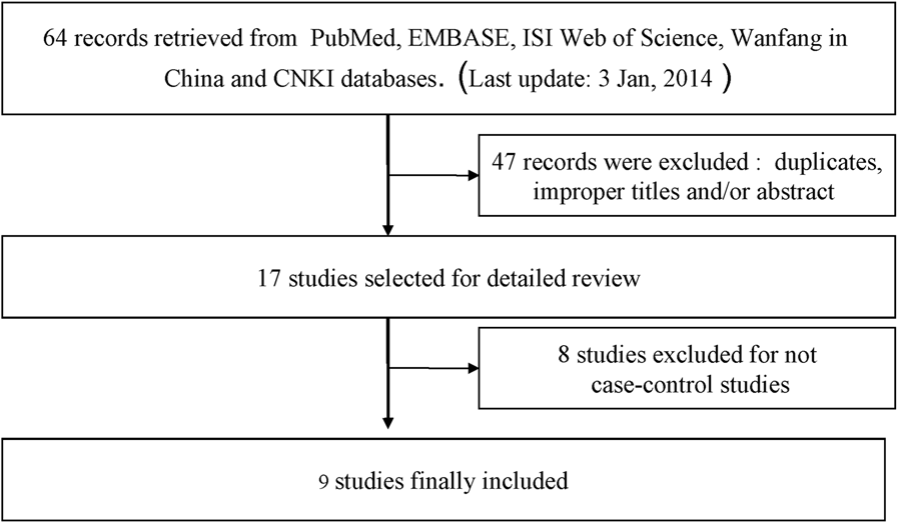
Flow chart of selection of literatures.

As shown in Figure 1, there are 98 literatures preliminarily detected, and 89 literatures were excluded due to duplicated publication, not providing genotype data and non-clinical based research literature. A total of 9 literatures were included. These 9 studies [17-25] including 4460 patients with type 2 diabetes and 4114 healthy control subjects were included in this research.

### The characteristics of these studies

As shown in Table 1, in these 9 studies, there were 3 studies carried out in Caucasian population, and 6 studies performed in Asian population. All these 9 studies provided genotype data and all are in line with Hardy-Weinberg equilibrium (HWE).

**Table 1:** The characteristics of included studies

**Authors**	**Publication year**	**Country**	**Ethnicity**	**Case/control (n)**	**Age**		**HWE**
					**Case**	**Control**	
Larsen et al. [24]	2005	Denmark	Caucasian	557/233	60 ± 11	52 ± 14	Yes
Kim et al. [23]	2006	Korea	Asian	206/80	56.4 ± 10.1	54.9 ± 9.5	Yes
Choi et al. [22]	2006	Korea	Asian	760 /641	59 ± 10	65 ± 4	Yes
Berthold et al. [20]	2009	Germany	Caucasian	420/430	61 ± 12	63 ± 7	Yes
Garcia et al. [25]	2009	France	Caucasian	610/820	60	52	Yes
Liu J, et al. [18]	2012a	China	Asian	864/877	53 ± 11	53 ± 12	Yes
Liu J, et al. [21]	2012b	China	Asian	744/1024	56 ± 8	54 ± 11	Yes
Xu et al. [19]	2008	China	Asian	333/202	50 ± 8	49 ± 6	Yes
Zhang et al. [17]	2011	China	Asian	138/113	54 ± 12	54 ± 15	Yes

### The relation between MTLRP polymorphism and Type 2 diabetes

**Figure 2:**
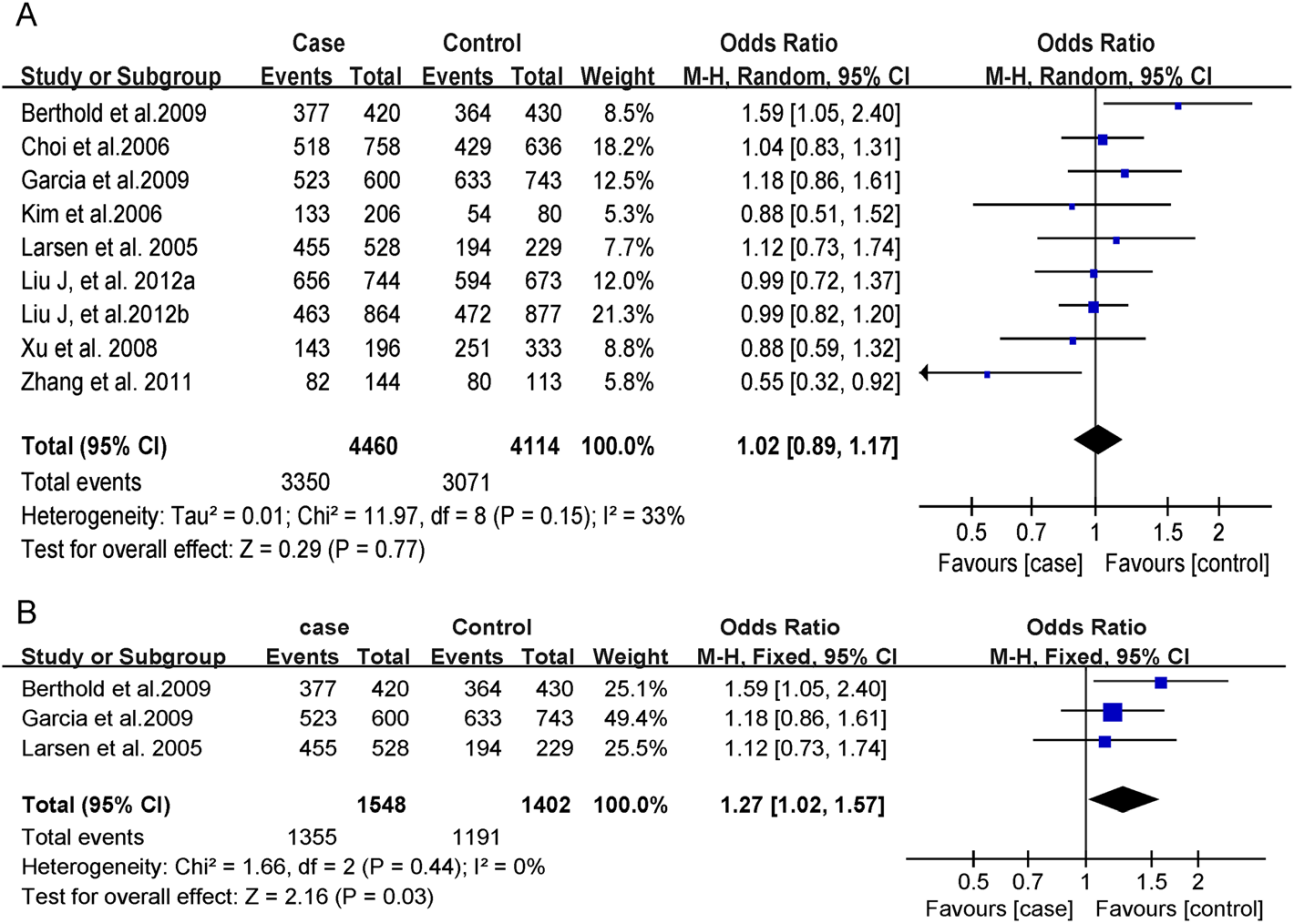
**Forest plot of MTLRP genetic polymorphism (214C>A) and Type 2 diabetes (CC genotype *vs *CA + AA genotype), the horizontal lines correspond to the study-specific OR and 95% CI, respectively.** The area of the squares reflects the study-specific weight. The diamond represents the pooled results of OR and 95% CI. (**A**: In total; **B**: In Caucasian).

**Figure 3:**
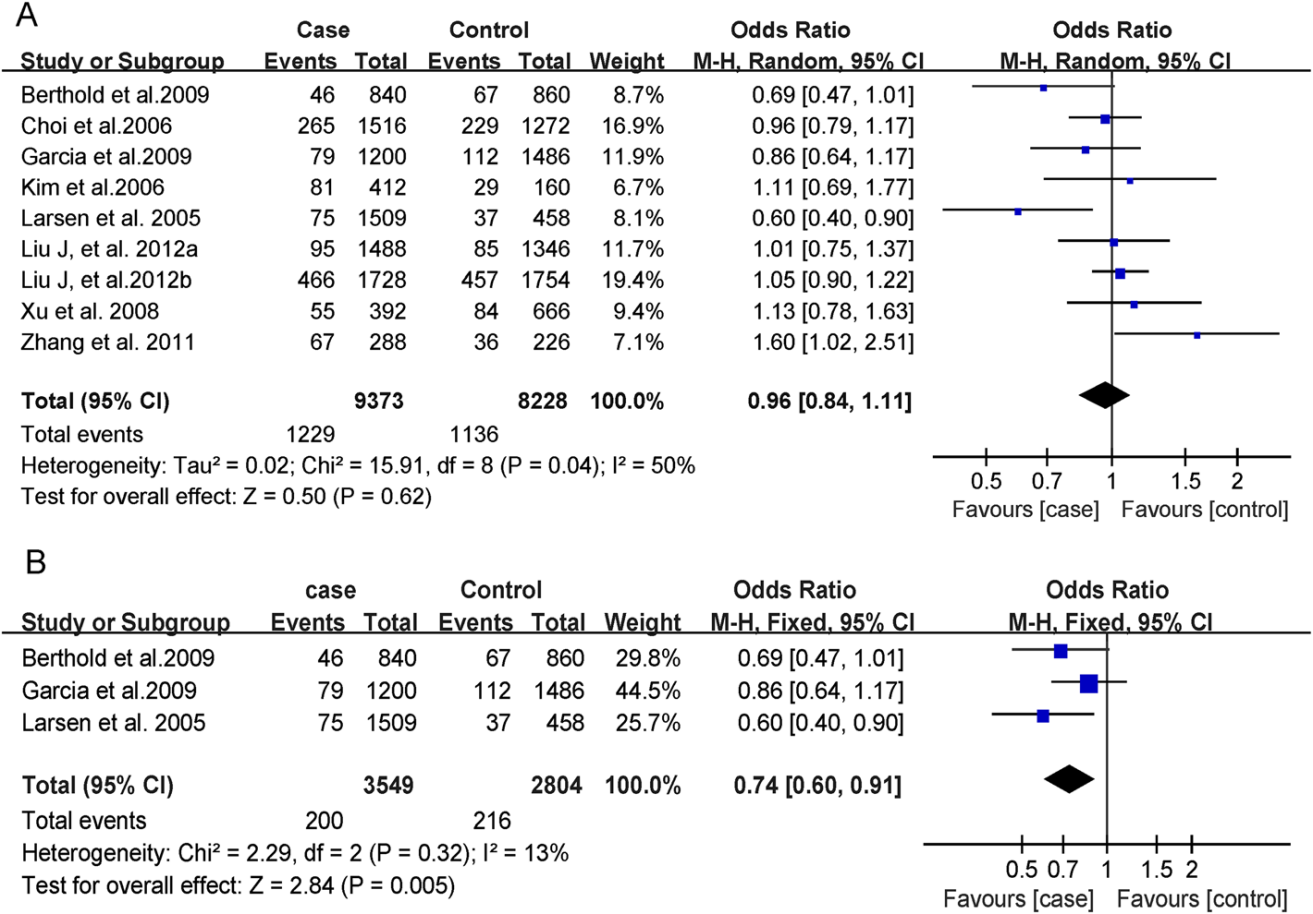
**Forest plot of MTLRP genetic polymorphism (214C>A) and Type 2 diabetes (A allele *vs *C allele), the horizontal lines correspond to the study-specific OR and 95% CI, respectively.** The area of the squares reflects the study-specific weight. The diamond represents the pooled results of OR and 95% CI. (**A**: In total; **B**: In Caucasian).

There was not significant heterogeneity between each study (P = 0.15, I^2^ = 33%) in overall population (P = 0.15, I^2^ = 33%) and in Caucasian population (P = 0.44, I^2^ = 0%). Therefore, we utilized the fixed-effect model to merge OR value. The meta-analysis results (Figures 2 and 3) suggested that, in overall population, there are not significant differences between Type 2 diabetes and healthy control subjects in genotypes (CC *vs* CA + AA: OR = 1.02; 95% CI: 0.89-1.17, P = 0.77) (Figure 2A) or alleles (A *vs* C: OR = 1.02; 95% CI: 0.84-0.96, P = 0.62) distribution (Figure 3A). However, in Caucasian population, subjects with A allele have decreased risk for Type 2 diabetes (OR = 0.74, 95% CI: 0.60–0.91, P = 0.005) (Figures 2B and 3B).

### Sensitivity analysis

We deleted one single study from the overall pooled analysis each time to check the influence of the removed data set to the overall ORs. The pooled ORs and 95% CIs were not significantly altered when any part of the study was omitted, which indicated that any single study had little impact on the overall ORs.

### Publication bias analysis

**Figure 4:**
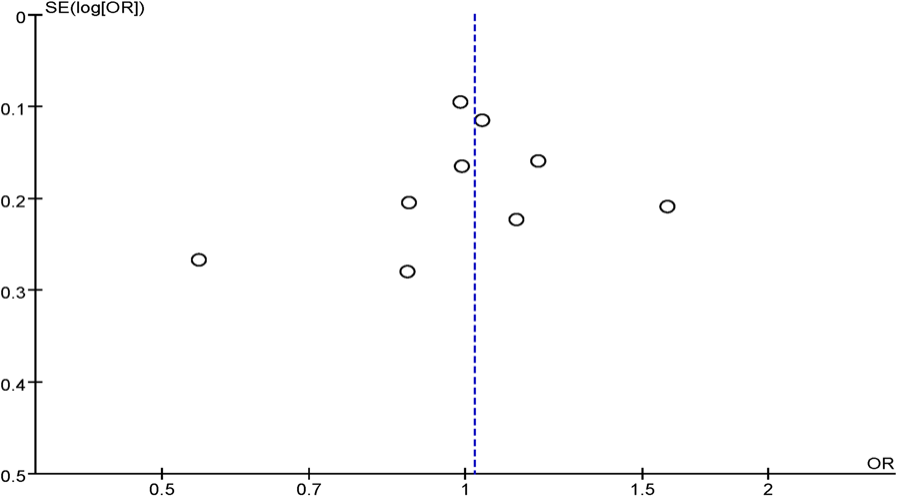
**Begg’s funnel plot for publication bias tests.** Each point represents a separate study for the indicated association. Log or represents natural logarithm of OR. Vertical line represents the mean effects size.

We analyzed publication bias by use of Revman 5.2 software, the funnel plot (Figure 4) shows the points evenly distributed, symmetrical, and most of the points are within the 95% confidence interval. It indicates there is no publication bias, and the result of study is credible.

## Discussion

In the present study, we found an association of *MTLRP* genetic polymorphism (214C>A) with Type 2 diabetes in Caucasian population. However, we did not found this association in Asian population.

Several previous studies suggested that *MTLRP* 214C>A polymorphism was associated with the risk for type 2 diabetes. However, other case–control studies reported conflicting results. This may partly be due to a small sample size in each of the published studies and ethnic difference. Meta-analysis is a useful statistical method that combines findings from independent studies. In the present study, we found that, in Caucasians, the risk for type 2 diabetes was decreased in subjects with C allele or CC genotype. However, this association was not found in Asians. The reason for this discrepancy may be as follows: 1) there are different genetic backgrounds between Caucasians and Asian, which plays an important role; and 2) type 2 diabetes is a sophisticated disease which is affected by an interaction between many factors, including environmental exposure, life style, socioeconomic status and individual susceptibility. It is possible that individual susceptibility in different ethnic group may be modified by environmental exposure, life style and socioeconomic status in a different way.

Further more, although we have found a positive association of MTLRP polymorphism with Type 2 diabetes in Caucasian population, the total sample size of Caucasians in this meta-analysis is still relatively small, which may restrict the statistical power for achieving a definitive conclusion. Therefore, case–control studies in larger samples are needed to confirm this correlation found in Caucasians.

In addition, the characteristic of meta-analysis is to combine comparable studies to increase the sample size and statistical power and draw a more compelling result. However, meta-analysis confounds factors such as publication bias, method of sampling, different genetic backgrounds of subjects, different protocols and quality of analysis. In the present study, we did not found the publication bias, and the genotypes in all studies were detected with genetic DNA from blood samples using PCR-RFLP genotyping methods. All of the studies checked genotypes for quality control. Genotype distribution of controls in all studies was consistent with HWE.

Exploring heterogeneity is one of the important goals of meta-analysis. In the present study no significant heterogeneity was found among the included studies. Sensitivity analysis also showed that omission of any single study did not have significant impact on the combined ORs. This made the results of this meta-study more reliable to some extent.

However, there remained some limitations in this meta-analysis. Although the genotyping methods used in all the studies were the same, other clinical factors such as age, sex and different treatment in each study might lead to bias. Determining whether or not these factors influence the results of this meta-analysis would need further investigation. In addition, although we concluded that there is no bias in our study by statistical analysis, in reality we all know that the papers having negative results were probably published with more difficultly. Therefore, the inevitable publication bias may exist in the results.

## Conclusion

Our study suggested that 214C>A polymorphism in the *MTLRP* gene was associated with risk of Type 2 diabetes in Caucasian population. Larger well-designed epidemiological studies with ethnically diverse populations and functional evaluations are warranted to confirm our findings.

## Competing interests

The authors declare that they have no competing interests.

## Authors’ contributions

LLC and SMH conceived the study, participated in the design, collected the data, performed statistical analyses, and drafted the manuscript. FFT and QL conceived the study, participated in the design, and helped to draft the manuscript. All authors read and approved the final manuscript.
